# OpenHiCAMM: High-Content Screening Software for Complex Microscope Imaging Workflows

**DOI:** 10.1016/j.isci.2018.03.017

**Published:** 2018-03-27

**Authors:** Benjamin W. Booth, Charles McParland, Keith Beattie, William W. Fisher, Ann S. Hammonds, Susan E. Celniker, Erwin Frise

**Affiliations:** 1 Biological Systems and Engineering Division, Lawrence Berkeley National Laboratory, Berkeley, CA 94720, USA; 2 Computer Science Research Division, Lawrence Berkeley National Laboratory, Berkeley, CA 94720, USA

**Keywords:** Optical Imaging, Technical Aspects of Cell Biology, Bioinformatics

## Abstract

High-content image acquisition is generally limited to cells grown in culture, requiring complex hardware and preset imaging modalities. Here we report an open source software package, OpenHiCAMM (Open Hi Content Acquisition for μManager), that provides a flexible framework for integration of generic microscope-associated robotics and image processing with sequential workflows. As an example, we imaged *Drosophila* embryos, detecting the embryos at low resolution, followed by re-imaging the detected embryos at high resolution, suitable for computational analysis and screening. The OpenHiCAMM package is easy to use and adapt for automating complex microscope image tasks. It expands our abilities for high-throughput image-based screens to a new range of biological samples, such as organoids, and will provide a foundation for bioimaging systems biology.

## Highlights

• OpenHiCAMM is an open source software that integrates robotics and image acquisition
• OpenHiCAMM is a module for the Fiji/ImageJ and μManager software packages
• OpenHiCAMM tracks images on multiple slides and controls an optional slide loader
• OpenHiCAMM is easily adapted for high-throughput screening and image analysis

## Graphical Abstract

**Figure:**
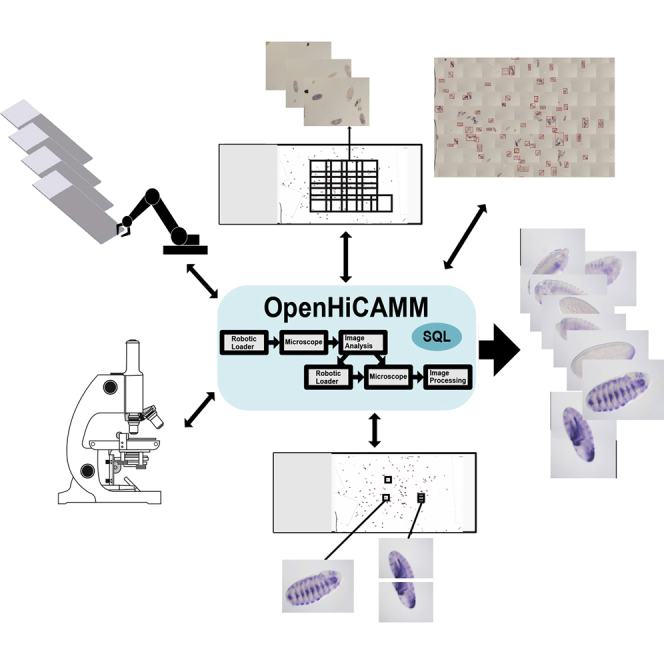

## Introduction

Large-scale genome sequencing has rapidly facilitated the investigation and analysis of gene and protein functions and interactions. Efforts to interpret sequence data and to understand how they are used to control cellular, tissue, or organ system development have quickly revealed the limitations in our molecular understanding of multicellular organisms. Spatial gene expression is important for understanding the events that are necessary for the development of metazoans, and large-scale studies are underway for a number of species (e.g., Hammonds et al., 2013, Lein et al., 2007, Pollet et al., 2005, Tabara et al., 1996). Indeed, even the existence of uniform cells or tissues has been questioned, and their variance has been masked by single measurements (Levsky and Singer, 2003).

High-content screening (HCS) is routinely used in cell culture imaging, pharmaceutical drug discovery, genome-wide RNA interference (RNAi), and CRISPR (Boutros et al., 2015). Commercial equipment is readily available (Zanella et al., 2010) and able to scan multi-well plates and slides. Commercial automated microscopes are integrated packages of robotics, microscope, and software, limiting possible customizations, especially for well-suited existing microscope systems. Imaging is done in a single pass, requiring a compromise between resolution and field depth. For single cell samples, this means a tradeoff between resolution and cell density. Transient transfections may not have sufficient cell transfection density for high-resolution imaging. For larger samples, such as histological sections, organoids, or whole-mount samples of model organisms or tissues, specimens need to be tiled at low resolution or placed at a predefined position.

We describe new software for high-throughput imaging, specifically designed to automate image acquisition that requires multi-step workflows contingent on image analysis and multiple imaging modalities. The software, OpenHiCAMM (Open Hi Content Acquisition for μManager) controls optical microscopes and interfaces with an automated slide loader to perform fully automated HCS.

## Results

We developed OpenHiCAMM as a module for the popular open source bioimage analysis package Fiji (Schindelin et al., 2012) and microscope hardware control μManager (Edelstein et al., 2010). OpenHiCAMM utilizes μManager for its broad support of microscopes, components, and cameras and its flexible slide acquisition. For advanced image analysis, our modules use Fiji's software components. Other existing μManager extensions are designed for thorough exploration of single samples (Pinkard et al., 2016, Pitrone et al., 2013), whereas our software is designed to process large sample sets.

**Figure 1:**
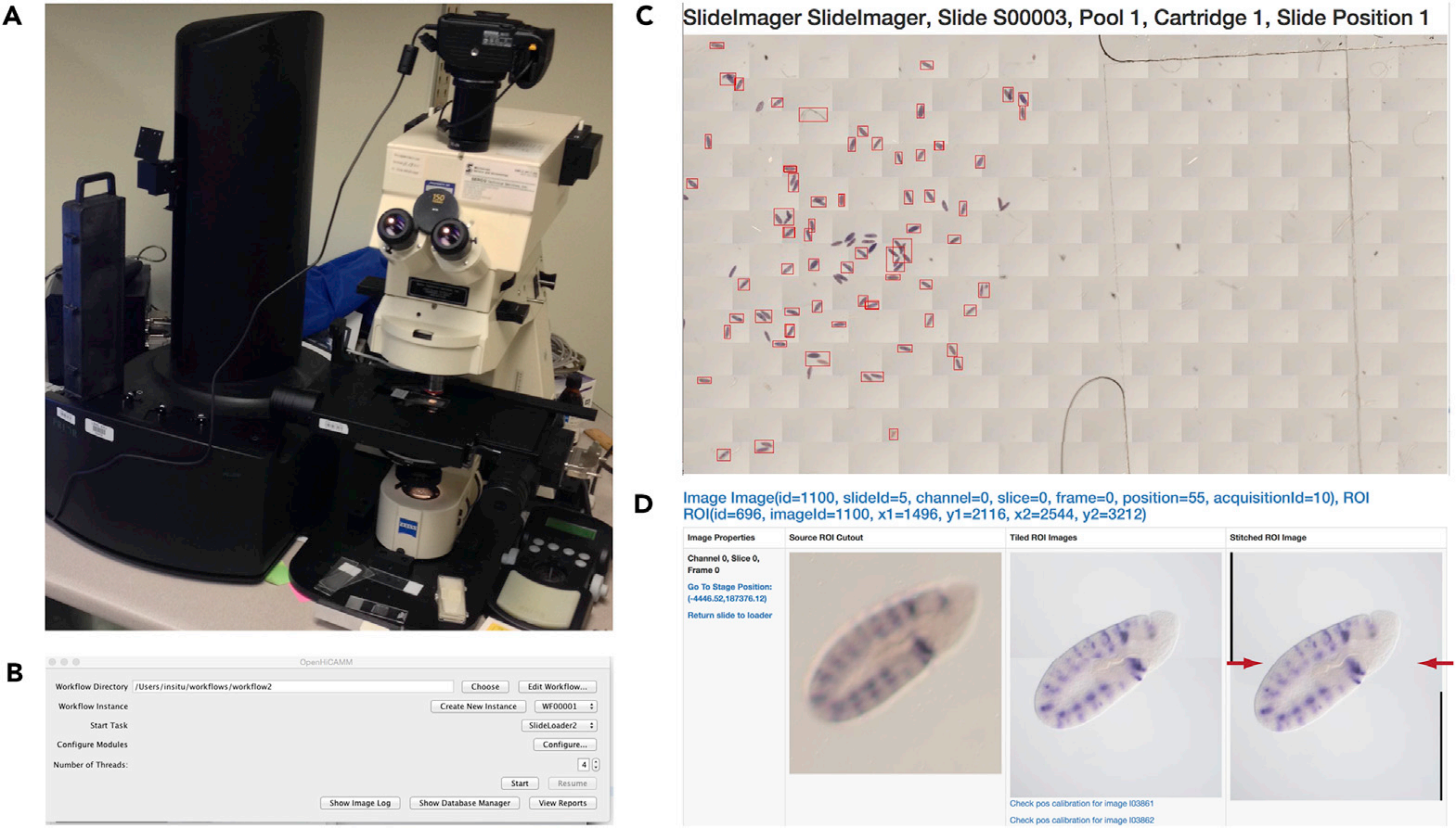
Microscope Setup and OpenHiCAMM Results (A) Microscope with motorized stage (on the right) and the automated slide loader (on the left). (B) Main dialog box for OpenHiCAMM, showing the workflow storage directory in use, tabs allowing user configuration options and access to reports and logs. See also Figure S10–S14. (C and D) Excerpts of the OpenHiCAMM report. (C) Image of a processed slide, stitched by stage position and the automated detection of regions of interest (ROI) shown boxed in red. (D) Screenshot from the report page showing the results for one detected ROI; (left) the image at 5× magnification, (middle) the image at 20× magnification superimposed by relative position, (right) the final image at 20× magnification stitched with the Fiji's algorithm (red arrows indicate the stitching position). See also Figure S1.

The core of OpenHiCAMM is a sophisticated workflow manager that executes modules operating the robotic hardware, performing the imaging and processing the data (Table S1, Figure S3 and Tables S2–S4). In the spirit of Fiji and μManager, we designed a high-quality, open and extensible Java core. The workflow manager executes microscope-dependent modules sequentially and computational processing modules in parallel. OpenHiCAMM uses an integrated SQL database engine for persistent storage of workflow setups, for module configurations, and for recording completed tasks, thus allowing for recovery from operational hardware problems and stopping or resuming interrupted workflows. Both the database files and the image files, in standard μManager file and directory format, are stored in a user-selectable local or remote disk destination (Figure 1).

To automate slide handling for HCS, we developed a slide management module, SlideLoader, which tracks the slides and either interfaces with a hardware slide loader or, for semi-automatic imaging without the slide loader, prompts the user to place the slide on the stage. SlideLoader can be used multiple times in the same workflow, allowing for loading and imaging slides repeatedly. This design allows for changing image modalities and manual adjustments of the microscope between each imaging pass. Loading slides multiple times may result in possible offsets to the slide position on the stage. To correct for these offsets, we developed modules for calibrating the stage position using the location-invariant edge between the frosted end and the adjacent transparent glass in commercially available slides (Figures S4 and S5).

Imaging of the slide is performed by the SlideImager module, which is a wrapper for μManager's Multi-D acquisition and thus able to use all capabilities of μManager, including selectable light filters and stacks along the z axis. If an area exceeds the size of a single camera snapshot, multiple tiled images are acquired and post-processed with Fiji image stitching to assemble a composite image (Preibisch et al., 2009) (Figure 1D). To increase the acquisition rate, we developed a fast custom autofocus plugin (Figure S6).

In our initial implementation, we developed a workflow and modules optimized for imaging slides containing whole-mount *Drosophila* embryos with gene expression patterns detected by *in situ* mRNA hybridization (Weiszmann et al., 2009). We assembled an HCS microscope platform with a Prior PL-200 slide loader, a Zeiss Axioplan 2 motorized microscope, a Nikon DSLR D5100 camera, and a Prior motorized stage (Figure 1B). We selected differential interference contrast (DIC) microscopy for capturing both probe staining and sample morphology in a single image, thus making it particularly well suited to high-throughput analysis of tissue or organisms and for studying ontogeny, such as the developing *Drosophila* embryo (Hartenstein and Campos-Ortega, 1997). We designed the workflow to image *Drosophila* embryos with two imaging modalities (Figure S9). For the first pass (Phase 1), we calibrated the microscope for a 5× objective. We ran the workflow to first image manually selected areas of the slide occupied by the coverslip as tiled images, followed by an image analysis module to capture the coordinates of the detected embryos (Figures S7 and S8). After completion of the first pass, we manually adjusted the microscope, moving to a 20× objective, and resumed the workflow at the second entry point (Phase 2). The workflow re-loads the slides and re-images the detected embryos at higher magnification.

To assess the performance, we measured the precision and speed of the imaging process. We manually created imaging objects on slides with a permanent marker, performed the imaging workflow, and manually superimposed the objects. The average displacement was 0.059 μm, about 1.5% of the image at 20× magnification (Figure S5). On average, the tiling at 5× took 2.3 s/image and the detection of twenty-five 20× images, including a focus step, took 12 s/image. At 5× magnification, the slides can be covered with 200–300 tiles, resulting in a rate of about 12 min/ slide. The second-phase high-resolution imaging with 20× magnification takes about 20 min for each 100 detected objects. Imaging the slide with high resolution at 20× would take over 3,000–6,500 images. Thus with approximately 300 embryos per slide, our imaging strategy achieved a more than 10-fold speedup.

To demonstrate OpenHiCAMM's ability for autonomously completing an HCS experiment, we used the workflow to image 95 slides made from a 96-well plate experiment (Figure S1). For the low-resolution pass, we selected a slide area with 180 tiles. Low-resolution imaging was completed in about 12 hr or at a rate of 8 min/ slide and yielded 26–751 objects (continuous areas containing one or multiple embryos) per slide. In the second pass, we obtained high-resolution images for embryos with imaging times ranging from 39 min (61 objects with 119 images) to 113 min (334 objects within 573 images) for 90% of the slides excluding those at the tails of the distribution (too few or too many embryos per slide).

For cases that rely only on high-resolution imaging, we developed an additional module, SlideSurveyor, which takes advantage of the camera video feed to rapidly image the slide from live view. We detect objects and re-image with SlideImager. All steps use the same imaging modality, thus limiting alignment problems and user intervention from repeated slide loading. Using SlideSurveyor for Phase 1 at 20× magnification resulted in 20 min/slide, while avoiding slide reloading and changing the objective.

**Figure 2:**
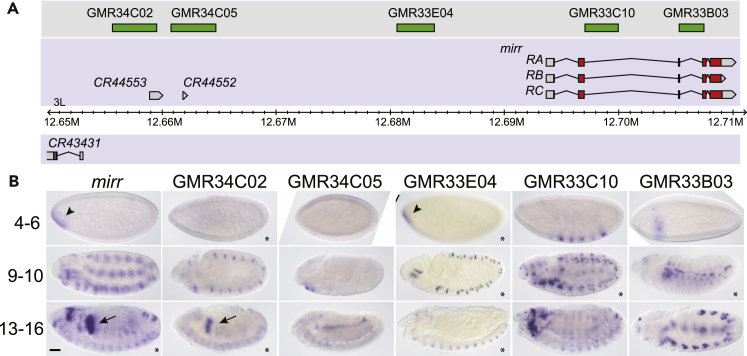
*Drosophila* Embryonic Images Acquired with OpenHiCAMM (A and B) (A) Genomic map of the *mirror (mirr)* locus. (B) Expression of the *mirr* gene in embryonic stages 4–6 (blastoderm), 9–10 (gastrulation), and 13–16 (terminal differentiation) visualized by whole-mount *in situ* hybridization with a probe to *mirr* mRNA shown adjacent to the expression produced by the fragments GMR34C02, GMR34C05, GMR33E04, GMR34C02, and GMR34C05. Transgene expression is visualized by whole-mount *in situ* hybridization with a probe to *GAL4* mRNA. Lateral views are shown, anterior to the left. Expression in the foregut anlage in statu nascendi (AISN) is indicated by arrowheads, and expression in the proventriculus is indicated by arrows. Segmental expression apparent at stage 9–10 in the wild-type *mirr* embryos is detectable at stage 4–6 in GMR33C10. Eight images (marked with an asterisk) are composite stitched images from overlapping tiled ROIs. Scale bar, 50 μm. See also Figure S2.

We imaged six additional slides to compare the expression of *Drosophila* embryonic wild-type gene *mirror (mirr)* (McNeill et al., 1997) with two intragenic and three intergenic cis-regulatory module (CRM) reporter constructs (Pfeiffer et al., 2008) (Figures 2 and S2). For high-quality slides, our workflow acquired between 75% and 85% of the objects on the slide. One slide (GMR33E04) exhibited age-related degradation (low contrast) and detected only 55% of the total objects on the slide. For each slide, we obtained high-quality images representing six stage ranges and two standard orientations as previously described for manual imaging (Hammonds et al., 2013). The images obtained from slides containing embryos with CRM constructs were compared with the images obtained from slides containing wild-type embryos. The collected images were of sufficiently high resolution to allow identification of distinct elements of the wild-type pattern driven by different CRMs (Figures 2B and S2). These results were comparable with those from similar experiments performed using manual imaging (Pfeiffer et al., 2008).

## Discussion

Imaging multicellular samples has become increasingly essential for understanding the complexity of medically relevant biology. To our knowledge, no existing tool is capable of multiple-slide autonomous imaging or adapting studies focused on multicellular samples to HCS. OpenHiCAMM adds the abilities to (1) accommodate hardware beyond the microscope itself as demonstrated with the slide loader, (2) handle hardware and software workflows, and (3) track slide contents beyond a single plate/slide setup. To accomplish these goals, we developed a sophisticated task algorithm, capable of resolving hardware and software task dependencies and providing persistence across workflows or interrupted experiments with an SQL database back end. These advances provide the flexibility to adapt workflows currently used for HCS, and material science samples, as well as allow integration of experimental devices, such as flow cells activated by changes computationally recognized in an acquired image.

Imaging cells in 96-well plates is well supported, both commercially and by open source products (Singh et al., 2014). OpenHiCAMM expands HCS to sample types for which there is no current solution while providing a robust, self-contained package and a transparent user interface, similar to the Fiji bioimaging package and commercial imaging and microscopy software (e.g., Opera/Operetta by Perkin Elmer). Previously existing tools are implemented as macro packages with only a subset of our software's capabilities (Edelstein et al., 2010), or are dependent on external software such as LabView (Conrad et al., 2011). As an important advance over a previous workflow design (Eberle et al., 2017) implemented in KNIME (Berthold et al., 2009), our software enables experiments for a high-content screen on multiple slides or multi-well plates. The commercial Metafer Slide Scanning platform is closest in capabilities but is dependent on a single hardware setup and is solely aimed toward histology content at lower resolutions. Magellan utilizes μManager and serves as a complementary utility for exploration of single-slide 3D samples or could conceivably be adapted as powerful plugin to our software.

Object recognition has been the primary focus of previous works (Conrad et al., 2011, Eberle et al., 2017, Tischer et al., 2014) but is only a relatively insignificant part of our software. We tried to avoid the domain specificity of machine learning by creating an intentionally simple but more generalizable object recognition algorithm, while allowing users to add their own recognition pipeline developed in ImageJ/Fiji and pasted as a macro. More sophisticated machine learning algorithms are easily added with a simple programming interface. In fact, the well-supported KNIME software (Berthold et al., 2009) would make a highly complementary plugin to our software for complex image processing workflows to detect specific features in biological samples.

Our workflow framework is robust, flexible, easily adapted, and extendable to other high-throughput robotics tasks that are increasingly common in modern biology, making the OpenHiCAMM package uniquely positioned to provide a foundation for sophisticated high-throughput screens.

## Methods

All methods can be found in the accompanying Transparent Methods supplemental file.

## Supplemental Information

Document S1. Transparent Methods, Figures S1–S14, and Tables S1–S4

## References

[bib1] Berthold M.R., Cebron N., Dill F., Gabriel T.R., Kötter T., Meinl T., Ohl P., Thiel K., Wiswedel B. (2009). KNIME - the Konstanz information miner: version 2.0 and beyond. SIGKDD Explor. Newsl..

[bib2] Boutros M., Heigwer F., Laufer C. (2015). Microscopy-based high-content screening. Cell.

[bib3] Conrad C., Wunsche A., Tan T.H., Bulkescher J., Sieckmann F., Verissimo F., Edelstein A., Walter T., Liebel U., Pepperkok R. (2011). Micropilot: automation of fluorescence microscopy-based imaging for systems biology. Nat. Methods.

[bib4] Eberle J.P., Muranyi W., Erfle H., Gunkel M. (2017). Fully automated targeted confocal and single-molecule localization microscopy. Methods Mol. Biol..

[bib5] Edelstein A., Amodaj N., Hoover K., Vale R., Stuurman N. (2010). Computer control of microscopes using microManager. Curr. Protoc. Mol. Biol..

[bib6] Hammonds A.S., Bristow C.A., Fisher W.W., Weiszmann R., Wu S., Hartenstein V., Kellis M., Yu B., Frise E., Celniker S.E. (2013). Spatial expression of transcription factors in *Drosophila* embryonic organ development. Genome Biol..

[bib7] Hartenstein V., Campos-Ortega J.A. (1997). The Embryonic Development of *Drosophila melanogaster*.

[bib8] Lein E.S., Hawrylycz M.J., Ao N., Ayres M., Bensinger A., Bernard A., Boe A.F., Boguski M.S., Brockway K.S., Byrnes E.J. (2007). Genome-wide atlas of gene expression in the adult mouse brain. Nature.

[bib9] Levsky J.M., Singer R.H. (2003). Gene expression and the myth of the average cell. Trends Cell Biol..

[bib10] McNeill H., Yang C.H., Brodsky M., Ungos J., Simon M.A. (1997). Mirror encodes a novel PBX-class homeoprotein that functions in the definition of the dorsal-ventral border in the *Drosophila* eye. Genes Dev..

[bib11] Pfeiffer B.D., Jenett A., Hammonds A.S., Ngo T.T., Misra S., Murphy C., Scully A., Carlson J.W., Wan K.H., Laverty T.R. (2008). Tools for neuroanatomy and neurogenetics in *Drosophila*. Proc. Natl. Acad. Sci. USA.

[bib12] Pinkard H., Stuurman N., Corbin K., Vale R., Krummel M.F. (2016). Micro-Magellan: open-source, sample-adaptive, acquisition software for optical microscopy. Nat. Methods.

[bib13] Pitrone P.G., Schindelin J., Stuyvenberg L., Preibisch S., Weber M., Eliceiri K.W., Huisken J., Tomancak P. (2013). OpenSPIM: an open-access light-sheet microscopy platform. Nat. Methods.

[bib14] Pollet N., Muncke N., Verbeek B., Li Y., Fenger U., Delius H., Niehrs C. (2005). An atlas of differential gene expression during early *Xenopus* embryogenesis. Mech. Dev..

[bib15] Preibisch S., Saalfeld S., Tomancak P. (2009). Globally optimal stitching of tiled 3D microscopic image acquisitions. Bioinformatics.

[bib16] Schindelin J., Arganda-Carreras I., Frise E., Kaynig V., Longair M., Pietzsch T., Preibisch S., Rueden C., Saalfeld S., Schmid B. (2012). Fiji: an open-source platform for biological-image analysis. Nat. Methods.

[bib17] Singh S., Carpenter A.E., Genovesio A. (2014). Increasing the content of high-content screening: an overview. J. Biomol. Screen..

[bib18] Tabara H., Motohashi T., Kohara Y. (1996). A multi-well version of in situ hybridization on whole mount embryos of *Caenorhabditis elegans*. Nucleic Acids Res..

[bib19] Tischer C., Hilsenstein V., Hanson K., Pepperkok R. (2014). Adaptive fluorescence microscopy by online feedback image analysis. Methods Cell. Biol..

[bib20] Weiszmann R., Hammonds A.S., Celniker S.E. (2009). Determination of gene expression patterns using high-throughput RNA in situ hybridization to whole-mount *Drosophila* embryos. Nat. Protoc..

[bib21] Zanella F., Lorens J.B., Link W. (2010). High content screening: seeing is believing. Trends Biotechnol..

