# Supplementary material for: OpenHiCAMM: High-Content Screening Software for Complex Microscope Imaging Workflows
Source: iScience. 2018 Mar 27;2:136–40. doi: 10.1016/j.isci.2018.03.017 (PMC5993205; doi:10.1016/j.isci.2018.03.017)
Supplement: Document S1. Transparent Methods, Figures S1–S14, and Tables S1–S4 [file mmc1.pdf]

ISCI, Volume *2*

Supplemental Information

OpenHiCAMM: High-Content Screening Software for Complex Microscope Imaging Workflows

Benjamin W. Booth, Charles McParland, Keith Beattie, William W. Fisher, Ann S. Hammonds, Susan E. Celniker, and Erwin Frise

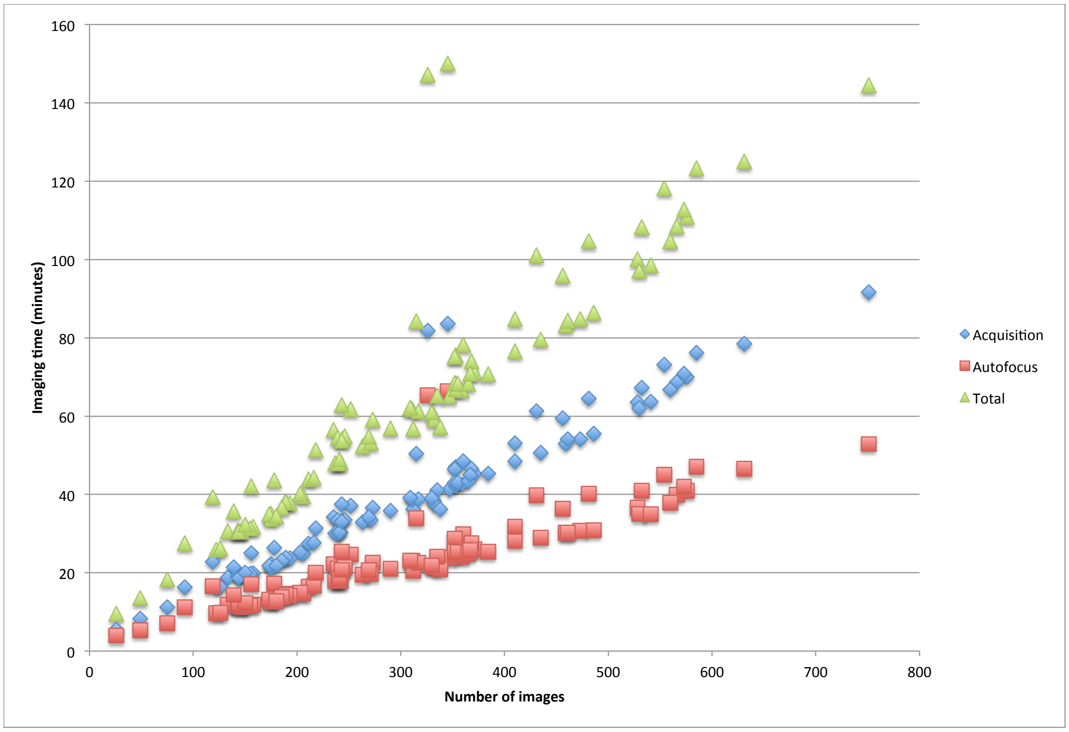

Figure S1. Times for imaging 95 slides from a 96-well experiment with Drosophila embryo samples, Related to Figure 1. The time in minutes is shown on the vertical axis, the number of images on the horizontal axis. The 90% percentile, i.e. 5% to 95% are between 119 and 573 images. Acquisition times vary according to the success of the autofocus process.

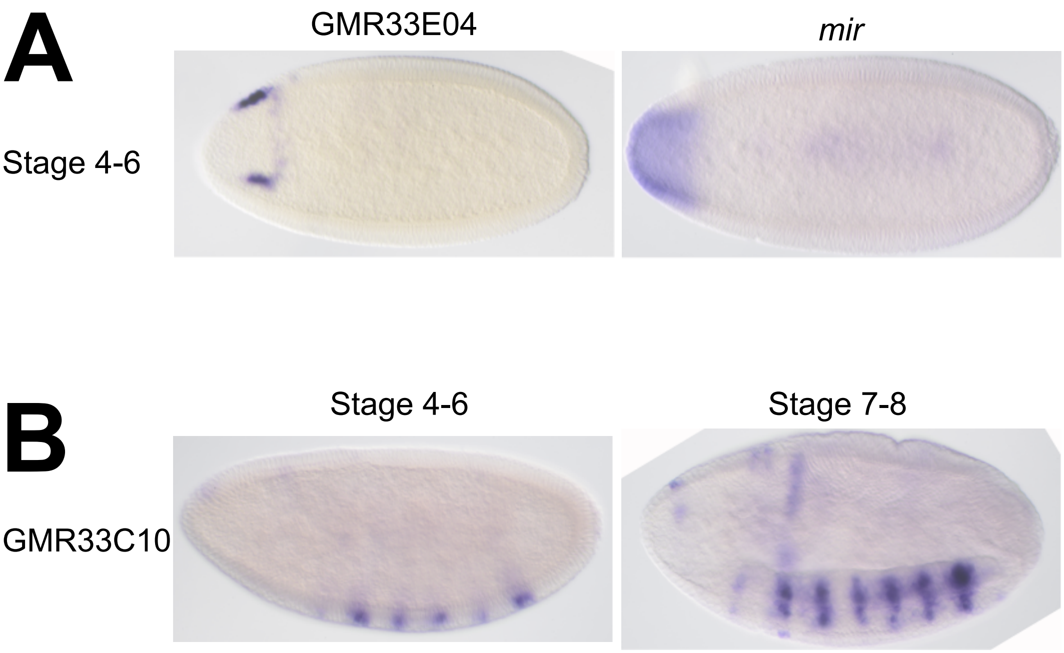

Figure S2. Additional supporting images for the *mirror* (*mir*) CRM experiment, Related to Figure 2. (**A**) GMR33E04 and wild-type mir expression shown at stages 4-6 from ventral. Wild-type exhibits a continuous pattern whereas GMR33E04 has two smaller expression domains at the boundaries of the wild-type gene expression. (**B**) GMR33C10 expression at stages 4-6 continues in a segmentally repeated pattern at stages 7-8.

Module name	Module function	Hardware control
**SlideLoader**	Slide management, loading slides or prompting user to place slide on stage	optional robotic slide loader
**SlideImager**	Imaging of a slide	Robotic stage, optional Z-axis and illumination/filters
**SlideSurveyor**	Fast low resolution imaging of a slide using live video feed from camera	Robotic stage
**BDGPROIFinder**	Region of interest detection	none
**CustomMacroROI Finder**	Region of interest detection with Fiji/ImageJ macros	
**ImageStitcher**	Registration and stitching of adjacent images	none
**refImager**	Imaging a reference position	Robotic stage
**compareImager**	Capturing set of tiled images around reference position defined in refImager	none
**PosCalibrator**	Computational comparison of reference images and comparison images and adjustment of position list.	none

Table S1. List of implemented modules and their functions for OpenHiCAMM, Related to Figure 1.

Transparent Methods

OpenHiCAMM workflow manager, data model and module management

OpenHiCAMM is written in the Java 1.8 programming language as a plugin for µManager 1.4.x. While compatible with the basic ImageJ and µManager distribution, for image processing, we require the extended and standardized plugin selection provided by Fiji. At its core is a custom workflow manager providing following functionality: 1) A common interface for modules implementing either hardware control or image processing tasks; 2) Configuration of a modularized workflow and module specific parameters; 3) Resolving dependencies and ordered execution of the modules; 4) Metadata and image storage management; 5) Response to events from the hardware; and 6) A graphical user interface for designing workflows, configuring storage and modules and starting/stopping and resuming workflows.

OpenHiCAMM itself is platform agnostic but µManager hardware support depends on the operating system. We developed and tested the C++ code for PL200 slide loader hardware adapter on Macintosh OSX 10.10 (or newer) also tested it under Linux.

The source code is available on Github (https://github.com/bdgp/OpenHiCAMM).

Data model

The OpenHiCAMM workflow engine manages two types of data, a set of execution tasks and the module and task configurations (Figure S3).

Execution tasks: Each task represents a single unit of work. The purpose of a task is 1) to allow the system to schedule module executions and 2) to provide a record of successful or unsuccessful module completion. Tasks are connected to each other using a directed acyclic graph. A workflow task may be started once all of its parent tasks have completed successfully. Workflow modules may tag tasks as serial or parallel. Serial tasks are completed in sequential order, and are suitable for hardware dependent tasks such as slide loading and imaging. Parallel tasks can be run simultaneously and are suitable for image processing or analysis. Each task consists of a unique task identification number, its current status, and its associated module. Tasks are connected together in parent-child relationships. The task workflow is similar to the concept of a data dependency graph that is common in many workflow execution systems (Zaharia, Chowdhury et al. 2010).

Workflow module and task configurations: Metadata for configurations are stored as key-value pairs. Workflow module configurations are parameters set by the user and can be accessed by all tasks in the module. Module configurations are used for configuring parameters that apply to all tasks within the module. Task configurations apply to each task individually. Task configurations are not manually entered by the user, but are generated by the module. The task configuration is used to store information about which item of work each task is to perform. For example, the SlideLoader task configuration stores the identification and location for the slide to load, the SlideImager task configuration stores the identification of the image to be acquired by that task.

Images are part of the task data and not explicitly handled. OpenHiCAMM directs the µManager acquisition engine to store images in its native format to a workflow dependent location and tracks imaging through the task sets.

Workflow implementation and API

System architecture

The OpenHiCAMM workflow engine is designed to store its data in a SQL database backend. Our current version implemented HSQLDB (http://hsqldb.org) as database backend. We chose HSQLDB because it is implemented in pure Java and can be distributed as a cross-platform JAR file, making installation using the Fiji update manager much easier.

Each workflow module can create a configuration entry and one or more tasks. Upon invocation of a configured workflow, a new workflow instance with a task list is generated, tasks linked as acyclic graph and task information stored in the database. The workflow manager iterates through all pending tasks and executes every task not completed and with no dependency on preceding tasks or competing for the microscope hardware.

Each workflow can have multiple distinct phases. Each phase is named by its initial module, the topmost module in the graph. The user selects which phase to execute in the workflow dialog. Providing multiple phases and allowing the user to select between them gives the user the opportunity to split a workflow into sections and perform any required manual adjustments between each phase. For example, in our workflow designs, we expose two phases; the first performs a low-resolution scan and searches for regions of interest. The second performs a high-resolution tiled imaging of each region of interest found in the first phase.

For tasks tagged as “Serial”, child tasks will inherit task configuration from parent tasks. This allows a form of communication between parent and child tasks in the same workflow phase. For workflows split into separate phases, it may be desirable to pass information from the first phase tasks to the second phase tasks. This cannot be done using task configuration since the second phase tasks are not directly related to the first phase tasks. For these cases, the user can create custom database tables and add identifying information to each record so that the second phase modules can find the associated data. We use this approach in our workflow to pass the position list produced by the region of interest finder in phase 1 of our workflow to the phase 2 imaging module.

Modules have been optimized for robustness and rapid processing and acquisition. For modules interfacing with hardware, we added abstractions and modifications to the call sequence to catch aberrant hardware behavior and communication errors. The module will attempt to correct problems or skip the current step before stopping and reporting errors the user. Our improvements vastly increased the robustness of the stock µManager software and resulted in requiring user interaction only for hardware problems.

Module API

OpenHiCAMM is designed to be easily extensible using modules. Custom modules need to implement OpenHiCAMM’s module interface. Our Module interface allows user-created modules to customize the behavior of their modules using at several key entry points (Table S2).

Each module can provide a custom configuration user interface (UI). For the configuration UI, a module developer needs to implement a configure method and return a Configuration object (Table S3). If the module is part of the workflow, the UI will be automatically displayed in the tabbed configuration dialog (Figure S10). The configuration object will be managed and stored in the database by the workflow manager.

To add a new module, a module designer needs to add a Java jar file in the openhicamm_modules/ directory. On startup, OpenHiCAMM will automatically detect and load all jar files in the openhicamm_modules/ directory, and the module will be available in the Workflow Design interface. Module designers are free to design their own database tables, or use the provided configuration object for storing configuration metadata. A thin, easy-to-use wrapper around the popular ORMlite SQL object relational mapping library (http://ormlite.com) is provided in the Connection and Dao classes.

Reporting interface

OpenHiCAMM includes a reporting interface, which the module designer can extend to provide custom workflow reports (Table S4). We have used this reporting interface to successfully detect and correct implementation bugs and hardware issues in our workflow designs. By clicking the "View Reports" button in the Workflow Dialog, the Report Dialog is displayed with a drop-down list of the reports available for viewing. Module designers can build custom reports by implementing the Report interface and including the report in their plugin JAR file. The report will automatically be detected and added to the list of available reports.

Reports are created by interfacing with the OpenHiCAMM workflow manager to query the state of the workflow, and producing HTML output for display. To produce HTML with a convenient Java based interface, we included a HTML templating library named "Tag". Our Report UI uses the JavaFX WebView component to display the HTML document. JavaScript code can be added to the HTML document. We used JavaScript to interface the report with the microscope hardware, to allow for loading a slide and positioning the stage to a previously imaged object. Once the document is created, it is stored in the workflow directory, and can be re-generated at any time by the user.

Example workflow module implementation for detecting objects of interest

We implemented an ROIFinder module that executes an image processing pipeline to return an ImageJ ROI list with objects of interest (see section "ROIFinder and *Drosophila* embryo detection"). We used our workflow, μManager and Fiji data structures to integrate the output of the ROIFinder in a second phase workflow:

The ROIFinder module creates a position list of ROIs and stores it in a custom SlidePosList database table. In the first phase, the SlideImager module processes user defined SlidePosList to determine which areas of the slide will be imaged. In the subsequent phases, SlideImager processes the most recently added SlidePosList records. SlideImager computes if a region of interest is larger than the size of a single image and creates a set of partially overlapping tile positions.

The position list JSON schema defined by μManager allows for custom properties to be added to each position in the position list. The ROIFinder module adds the "stitchGroup" custom

property to each position in the ROI tileset to group the individual tiles together. The SlideImager module looks for custom properties in the position list and converts them into task configuration records. Because task configuration is inherited from parent to child for serial tasks, the downstream ImageStitcher module will use the “stitchGroup” task configuration to determine which images need be stitched together.

Core modules

SlideLoader module SlideLoader is responsible for initializing the slide loader hardware, keeping track of which slides are loading and unloading slides to and from the stage. SlideLoader defines a high-level programming interface with hardware dependent libraries, similar to the current µManager model. We developed a sample hardware library for a Prior PL-200 slide loader that supports most microscopes and is able to hold and handle up to 200 slides.

SlideImager module SlideImager is a wrapper for the µManager Multi-D dialog and acquisition engine, primarily providing configuration storage in the SQL database and invoking the the acquisition engine with the previously configured parameters. The position list can be either manually predefined or the result of a previous workflow module, such as ROIFinder. To manually create a position list, we use the µManager position list user-interface to define a region on the slide. SlideImager will pass configured parameters from the Multi-D dialog and the position list to the Multi-D acquisition engine. All functions provided by the Multi-D dialog are available, able to use all abilities of the µManager, including selectable light filters and stacks along the Z axis. We added a custom hook to the Multi-D acquisition engine, linking back to SlideImager, to track the acquisition of each image and schedule post-acquisition image processing tasks.

Calibration of the slide position on the stage

We developed the PosCalibrator module to processes images from a dedicated instance of SlideImager (Figure S4). Both modules can be optionally inserted into the workflow, after the slide is loaded onto the stage. SlideImager records images at an invariant position and PosCalibrator matches the slide position to a previous reference image and adjusts a position list to detected shift.

After loading a slide the first time, using the standard SlideImager module (called refImager in the example workflow in Figure S9), the user creates a position list with on entry containing the approximate position of the area where the slide frosting and the corner intersect. At each slide, when the workflow reaches SlideImager, the user-defined area is imaged and stored as reference (Figure S4A). At workflows that load the slide again, optionally at different magnification, the user can create an instance of a modified SlideImager module with additional functionality for re-imaging the area in case of an interrupted workflow (called compareImager in Figure S9), followed by PosCalibrator. SlideImager images the same area again and the images are matched to previously acquired images in the module PosCalibrator, Figure S4B.

While sophisticated scale and rotation invariant methods have been described, they are generally slow or hard to implement. For PosCalibrator, we created a fast and robust pipeline based on Generalized Hough Transform (GHT) template matching. GHT matches the edge

contours between a template and a target image by counting matching pixels in an accumulator (Ballard 1981). For each boundary point of the template, we pre-computed an R-table with the coordinate differences $x_c - x_{ij}$ and $y_c - y_{ij}$ between a fixed reference x_c/y_c in the middle and each boundary point x_{ij}/y_{ij}. To account for rotation and scaling we also pre-computed scaled and rotated x/y boundary pairs and stored them in the R-table. For each entry in the R-table, we calculated the gradient $\phi(x)$. For each pixel in the search image, we matched the pixel gradient to ϕ and increased the a corresponding entry in an accumulator array. The accumulator entry with the maximum value corresponds to the position of the template in the search image.

We customized an existing ImageJ plugin, GHT (http://rsb.info.nih.gov/ij/plugins/ght/index.html), and used the code in a new plugin to encapsulate the pipeline to process and return matched image coordinates as ImageJ ROI. The pipeline converts images to grayscale, resizes them and uses the ImageJ IsoData algorithm find two thresholding values for the reference and all combined calibration images. The threshold values are used to generate binary images, the images undergo morphological processing to eliminate holes and errant pixels. From the set of calibration images the pipeline selects an image containing the both slide frosting and slide border at roughly equal proportions. The reference image and the selected calibration image are converted to edge contours (Figure S4C) for matching with the GHT algorithm (Figure S4D). The workflow module PosCalibrator calls the plugin and transforms the matched image coordinates to stage coordinates. GHT is scale and rotation invariant but, to further reduce running time, we adjust the image sizes to the same dimensions with user configurable parameters (e.g. 4 for matching 5x and 20x magnifications). Image pixel translations to stage movements were measured with the µManager Pixel Calibration plugin and the resulting values entered as configuration parameters and used to adjust the offset. We tested the modules both with DIC and fluorescent illumination. If no matches are found, a warning message is added to the log and imaging continued with no calibration.

We tested our system with manual markings and found negligible displacements (Figure S5). Our slide calibration works both with light and fluorescent microscope systems.

Slide Surveyor

The SlideSurveyor module is similar to the SlideImager in that it takes as its input a position list and acquires images but does not use Micro-Manager's Multi-D acquisition module. Instead, SlideSurveyor sets the camera to “Live Mode”, moving to each position in the list, acquiring a live mode image. The image is then copied into an image buffer representing the entire slide. “Live Mode” captures video, which allows quickly acquiring low-resolution images without triggering the camera's shutter. Running the acquisition in “Live Mode” allows the SlideSurveyor to quickly map out an entire slide, even at 20x magnification. SlideSurveyor can image the contents of an entire slide in 20 minutes, as opposed to several hours with the Multi-D module and individual images. The resulting images are low quality, but sufficient for ROI detection. The same “Live View” mode is also used to accelerate our custom autofocus engine. The ability to survey an entire slide using the 20x lens enabled us to avoid using a two-phase workflow, helping the workflow to complete more quickly, and providing more accurate positioning and centering of the ROIs when performing the second phase.

Autofocus

We created a plugin, FastFFT (Figure S6), using the µManager autofocus API. In FastFFT, we perform the Fast Fourier Transform (FFT) using the Mines Java Toolkit (http://inside.mines.edu/~dhale/jtk/) and apply a bandpass filter to the power spectrum. The logarithmic values between the two percentage radiuses are summed up and the process repeated for all images in the same stack. The image with the highest value is selected. Empirically we determined the best bandpass filter between 15 and 80 percent to place the focal point in the middle of the embryo (Figure S6C). The bandpass filter values can be adjusted in the configuration dialog. To accelerate the image acquisition process, our autofocus module changes the camera setting to live imaging with reduced image size.

We compared FastFFT to two µManager autofocus implementations, J&M and Oughtafocus, by measuring the plugin reported level of detail at multiple stage positions for 10 embryos. The FastFFT module performed as designed and returned highest scoring focal planes at the middle of the embryos (Figure S6A and S6C). The µManager functions returned optimal focal planes with more variability, depending on the stage of the embryo (Figure S6A). At early embryos, focal planes were visually indistinguishable and within 6um of each other, less than the thickness of the cell-layer surrounding the blastoderm. At later stages, the µManager functions tended to focus on the surface of the embryo (Figure S6C). FastFFT performed a magnitude faster than the µManager function (Figure S6B), an average of 40 seconds for scanning 100 positions, compared to 206 seconds (Oughtafocus) and 246 seconds (J&M). Moreover, FastFFT was more consistent in its speed.

To further speed up processing and cover a wide range of focal planes, we perform a coarse focusing with large interval steps and a broad range and second a finer granularity focusing with small interval steps around the previous best focal plane. Both intervals are user configurable as part of the graphical configuration dialog. We configured it to take 41 images at 10 µm intervals and 7 images at 3.3 µm intervals. To prevent hardware damage cased by a potential slow drift in measurements, we also added a configuration for an absolute maximum/minimum Z position that cannot be exceeded. The plugin has a configurable counter to skip focusing on a selected number of images. The counter is reset every time after two consecutive images match the same Z position after completing the autofocus function. This prevents erroneous focusing on occasional mis-detected objects such as air bubbles.

ROIFinder and *Drosophila* embryo detection

This module processes single images and returns regions of interest (ROI). It provides a high level interface that returns an object's bounding box and can be easily adjusted to custom image processing pipelines. We developed two computationally efficient ROIFinder implementations. BothWe developed two computationally efficient custom ROIFinder implementations and macro based implementation, CustomMacroROIFinder, which allows the user to simply paste a previously developed ImageJ macro into a dialog and execute it as part of the workflow. CustomMacroROIFinder is extremely flexible and along with the powerful Fiji UI and macro

recording abilities allows for the development of complex segmentation workflows by users less experienced with image processing.

The custom implementations segment the image, use Fiji's "Analyze Particles" function to detect and measure segmented areas and store the bounding boxes for areas exceeding a selectable minimum size. We set the Analyze Particles function to discard objects at the image boundaries. Virtually all objects missed in our *Drosophila* embryo imaging experiment were discarded for crossing image boundaries rather than failure to detect the object.

The simpler ROIFinder variant segments the object by automatic thresholding with IsoData and is suitable for uniform samples distinct from background such as fluorescent labeled samples.

For our work with *Drosophila* embryos we developed a variant that uses the texture, which is frequently inherent in biological samples (Figure S7). Our pipeline calls Fiji edge detection, applies a threshold on the edge image with a fixed, empirically determined value (13, out of 255 gray values) and performs morphological image operations (close, fill holes) to smoothen the areas. This pipeline will be suitable for histological samples and other whole mount specimens.

For the SlideSurveyor workflow, we used the CustomMacroROIFinder (Figure S8). We developed a macro that 1) removes horizontal and vertical lines that were a result of SildeSurveyor's image stitching with Fiji's FFT Bandpass filter plugin, 2) resize, 3) convert to a mask, 4) thresholds the image, 5) performs morphological operations and, 6) applies the "Analyze Particles" function as described before.

ROIFinder provides a standard API for a image processing pipeline as well as the ability to execute a macro and using Fiji's image processing plugins, programming and macro recording abilities, a customized pipeline can be easily implemented. More complex samples such as mutant embryos with altered developmental phenotypes can be detected and rapidly imaged at high resolution for computational analysis. We envision users building sample specific object detection pipelines, which can significantly increase the throughput if uninteresting objects can be discarded at low resolution.

Drosophila embryo imaging workflow

Our two phase workflow is shown in Figure S9. After loading the slides, our SlideImager module captures images with a given list of positions on the slide. The initial position list is configured by the user as the maximum area of interest and slides are imaged in overlapping tiles at 5x magnification. Captured images are processed with a module, ROIFinder, to detect objects and their coordinates stored in the database. Detected objects are stored as bounding boxes, transformed to the imaging area and the content of the bounding boxes imaged again at higher resolution (Figure S1 and S2). If a bounding box exceeds the size of a single image at 20x, multiple tiled images are acquired and post-processed with a module, ImageStitcher, using an Fiji stitching algorithm (Preibisch, Saalfeld et al. 2009) to assemble a composite image (Figure 1D, Figure S2).

The Fiji based stitching worked reliably for 2-3 images but failed frequently for large composites with 3 or more images. We found these large composites containing dense clusters of overlapping embryos and discarded the results.

User interface

The main window is available from the µManager plugin menu (Figure 1). The user can select a local or remotely mounted (NFS or CIFS) directory as location for the workflow database, settings and images.

Using a workflow editor, the user can add and arrange modules from an existing set to create a workflow (Figure S9). For our low/high magnification implementation, we created a combined workflow with two imaging phases. The first imaging phase arranges the SlideLoader, refImager, SlideImager and ROIFinder modules, the second imaging phase arranges compareImager, PosCalibrator, another instance of SlideImager and ImageStitcher. Modules for the second imaging phase will automatically detect and use results from the first imaging phase. When the workflow reaches a point with no child tasks, it will terminate.

The "Start Task" in the main window defines the entry point, in our implementation, either the first or second imaging phase.

Each workflow module can be configured with persistent settings in a configuration dialog called from the main window (Figure S10-S12). The SlideLoader module creates a pool of slides with the current slides in the robotic slide loader, allows for scanning the slides and adding meta-data information (Figure S10). The SlideImager module will call the µManager Multi-D dialog for configuration of the slide imaging task and save the settings in the workflow database (Figure S13). SlideImager's derived modules, refImager and compareImager, are configured similarly. refImager takes a reference image for calibration at a user defined position. compareImager captures an overlapping set of images centered around the user defined position of RefImager. Upon successful completion both refImager and the set of compareImager images are used by the PosCalibrator to identify the matching position (see Detection of the slide position) and adjust the position coordinates. The ROIFinder module can be configured to record objects of a user defined minimum size and tested on images in the configuration dialog (Figure S12). Testing the ROI finder opens a window with images for each intermediate processing step and the resulting bounding boxes as illustrated in Figure S7 and S8.

For each defined workflow, new instances are used for processing a pool of slides. Thus, the same basic configuration can be used for multiple slide pools. Image files are stored in µManager format in a folder set by the initial workflow configuration and sub-folders matching the workflow instance and workflow tasks, respectively.

The workflow progress is stored in a SQL database. For debugging or correcting unexpected problems, the Workflow Dialog includes a "Show Database Manager" button that opens a graphical database querying interface with the workflow database loaded. The OpenHiCAMM source also includes a "sqltool" command-line script which, when run in the workflow directory, opens a command-line SQL query tool, which is useful for debugging purposes.

Reviewing the results

During workflow processing, progress can be monitored with a live imaging view and a log window (Figure S13). Workflows can be stopped and resumed at defined checkpoints. Upon completion, results can be visualized in an HTML report.

At the conclusion of the pipeline, OpenHiCAMM generates a summary report, displaying the calibration images, and the results for all imaging passes. The report is generated as an html file (Figure S14) and rendered in OpenHiCAMM in a report window. In OpenHiCAMM, the report viewer allows for loading slides and positioning the stage to positions. The report file can also be independently opened and viewed in a standard web browser.

In situ hybridization experiments

We performed in situ hybridizations as previously described (Weiszmann, Hammonds et al. 2009). We collected and hybridized wild type embryos with an RNA probe made from a cDNA clone of mirror and used a probe that detects Gal4 reporter RNA to hybridize embryos of five *Drosophila* strains, GMR33C02, GMR33C05, GMR33E04, GMR33C10, GMR33B03, from the Janelia Farm Research Campus (JFRC) FlyLight Gal4 collection (Pfeiffer, Jenett et al. 2008), each carrying a putative mirror enhancer with a Gal4 reporter.

Embryos were mounted on slides and imaged with OpenHiCAMM. Resulting images were cut out and rotated with Adobe Photoshop for publication.

Supplemental Figures

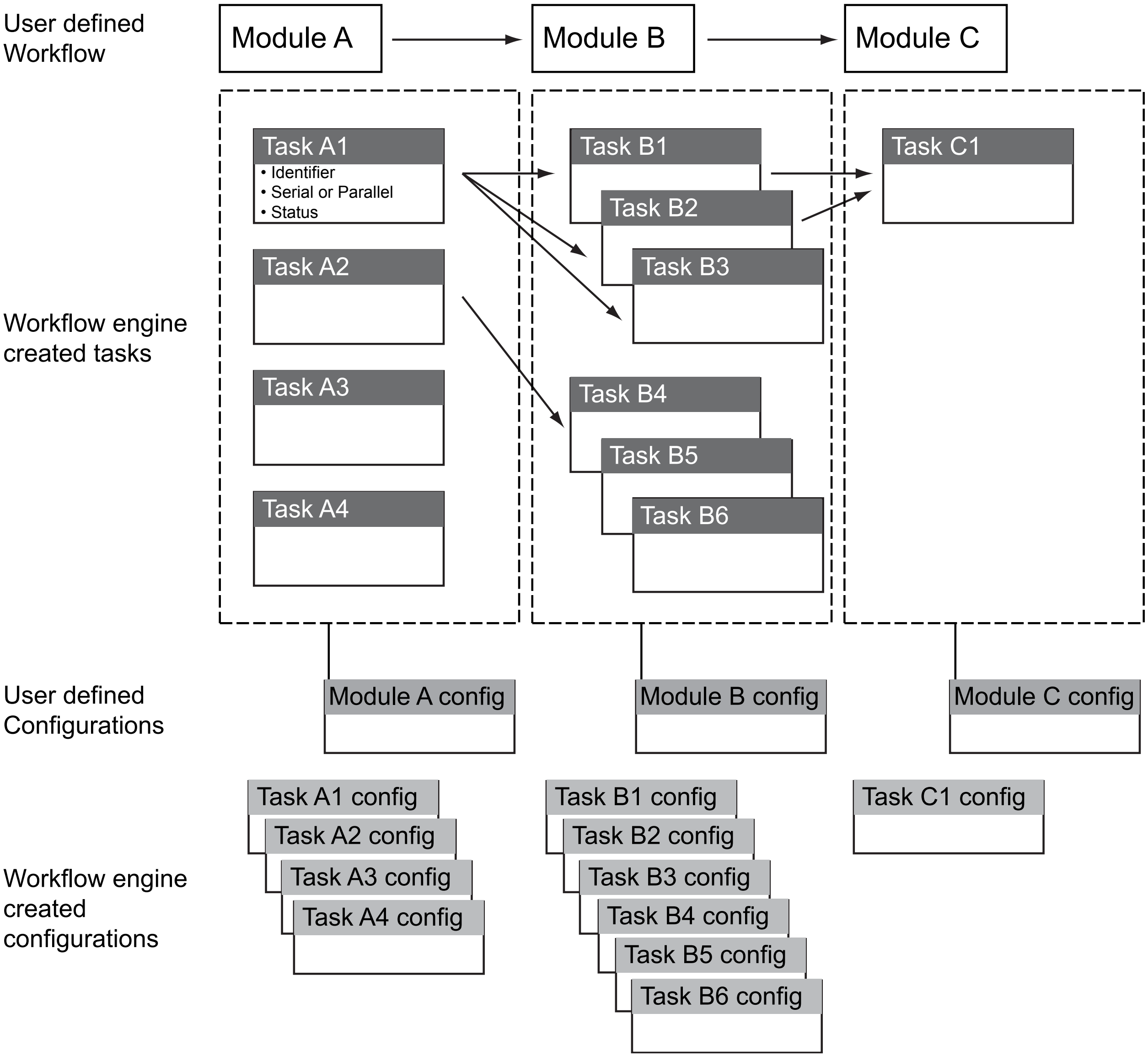

Figure S3. OpenHiCAMM data model, Related to Figure 1. Shown are three modules, labeled A, B, C with module specific tasks (labeled A1, A2, A3 for module A, etc.), module configurations and task configuration entries. The user defines the workflow and the module configurations, the workflow engine creates the tasks and the task configurations (left).

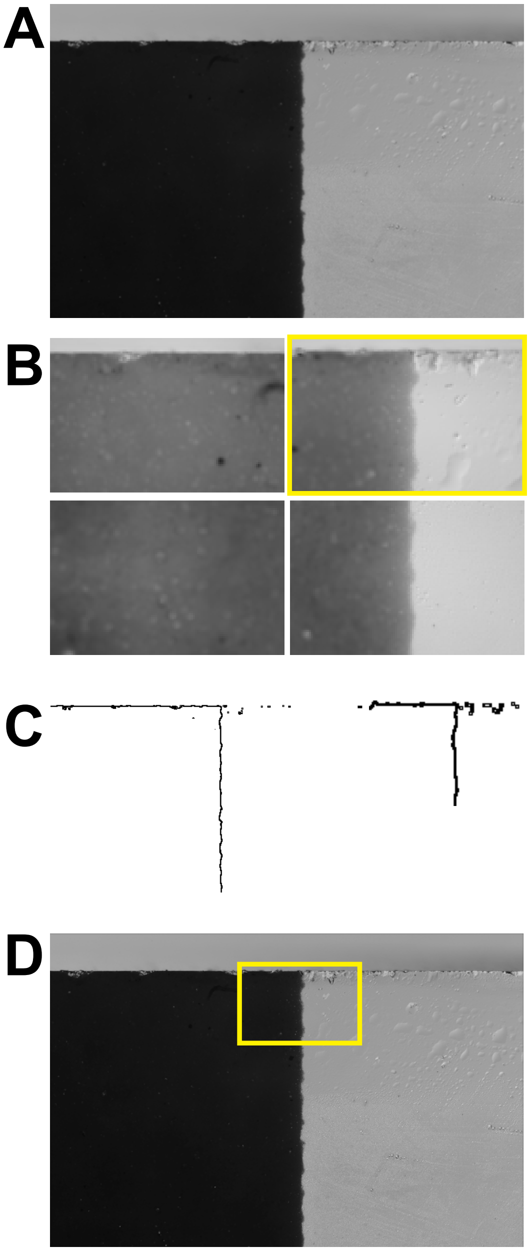

Figure S4. Alignment calibration for the same slide, loaded twice and imaged at different magnifications, Related to Figure 1. (**A**) Image at 5x magnification at position defined by the user. (**B**) Tiled images at 20x magnification around the original position of (A). The automatically selected calibration image is framed in yellow. (**C**) Edge images of (A) and (B) that are used for GHT. (**D**) Position of the calibration image in the reference image shown with a yellow box.

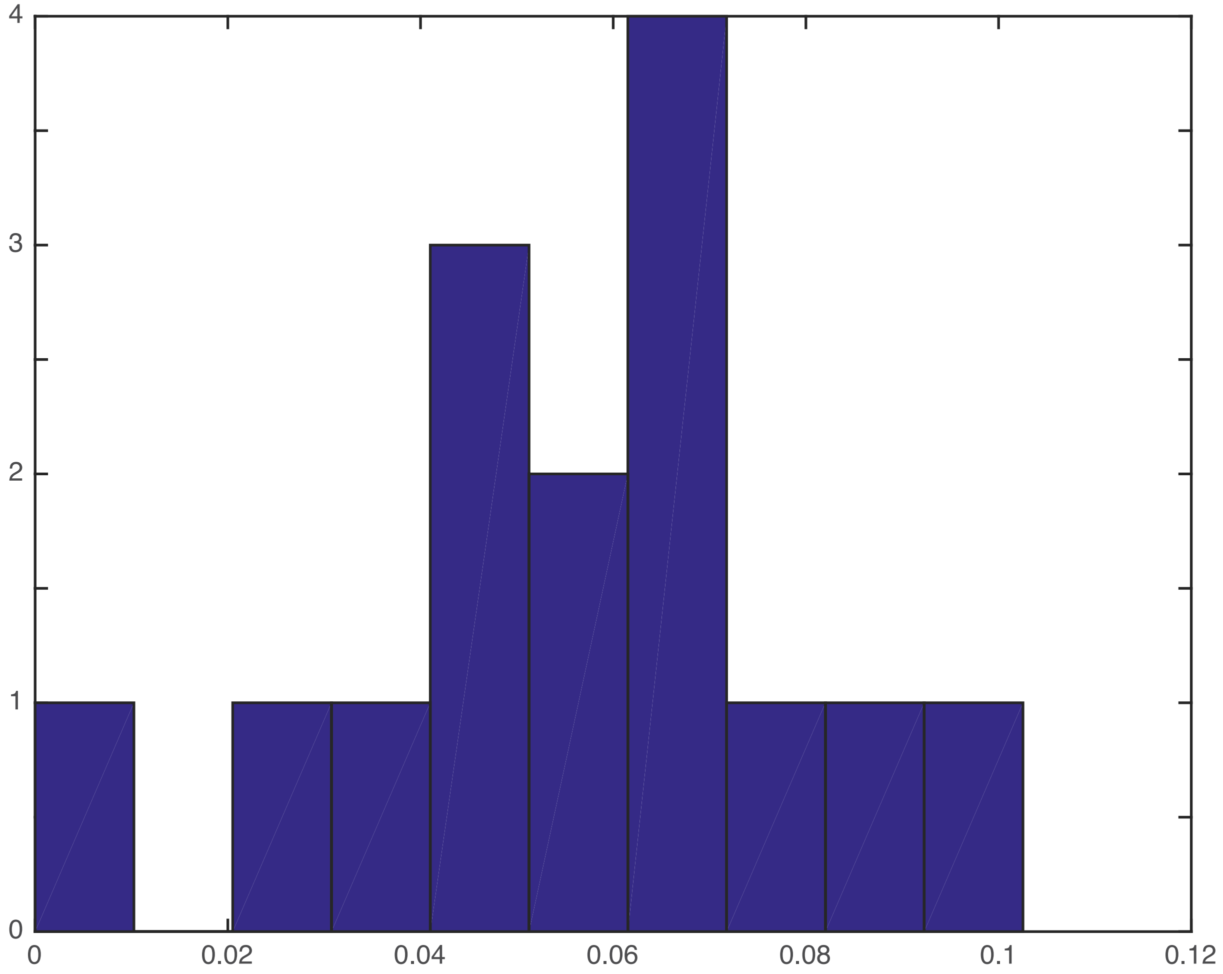

Figure S5. Precision of detecting same objects after calibrating slides, Related to Figure 1. Histogram depicts the euclidean distance between the position of 10 objects in μm.

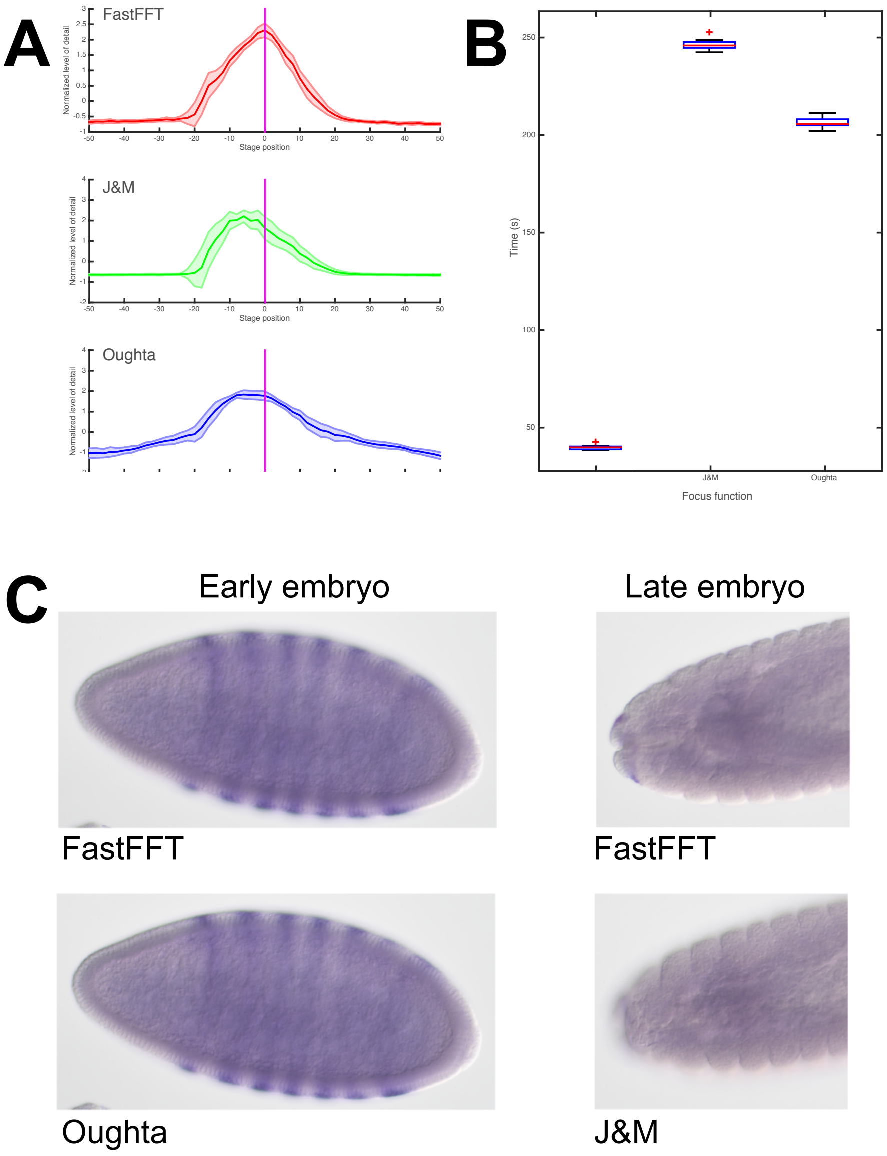

Figure S6. **Autofocus performance of FastFFT compared to µManager's J&M and OughtaFocus function (at default setting), Related to Figure 1.** (**A**) Median and standard deviation of normalized values returned from each plugin at stage positions -50um to +50um. Values reported by the autofocus functions were normalized as z-scores (centered to have mean 0 and scaled to have standard deviation 1). FastFFT is shown in red, J&M in green and OughtaFocus in blue. The stage positions for all three plugins are centered around the best value return for FastFFT. J&M shows the largest variance. (**B**) Box plot showing the execution times for the three plugins for the 100 sampled stage positions. (**C**) Early blastoderm embryo (left) and late embryo (right) showing the highest scoring focal planes for FastFFT (top) compared to OughtaFocus and J&M (bottom). Results show little discernible difference for the early embryo but a focal plane in the middle of the embryo for FastFFT compared to a focal plane on the surface for J&M.

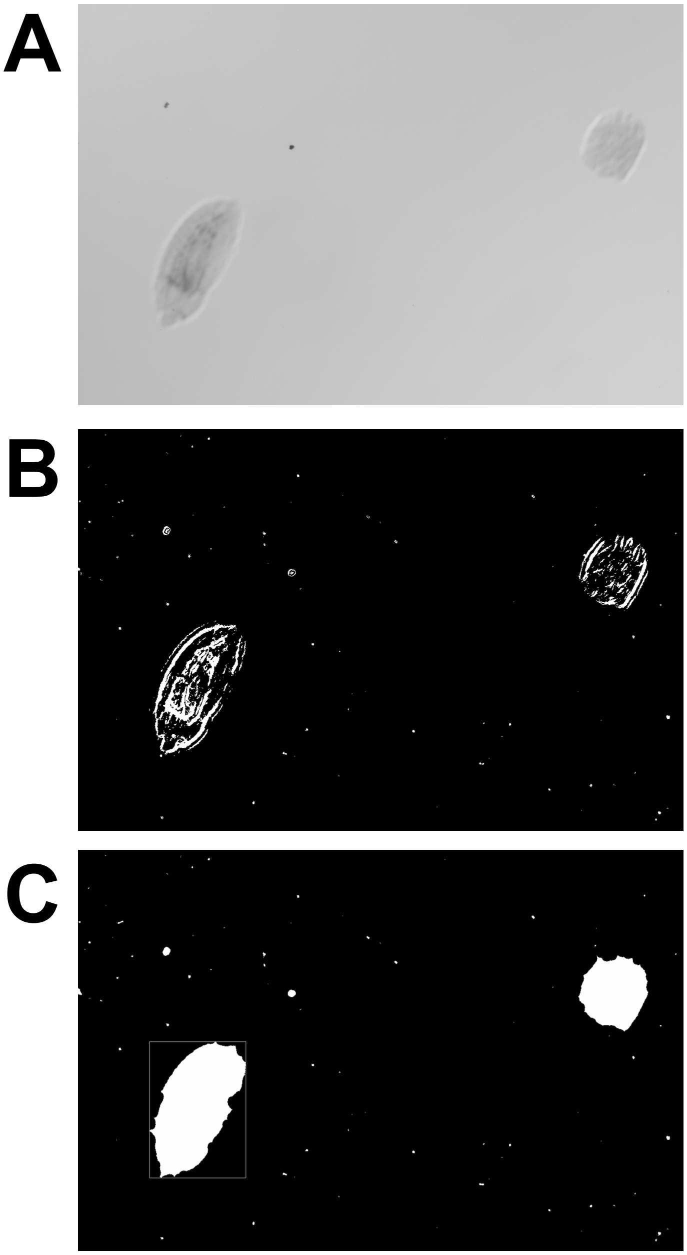

Figure S7. Visualization of results for the ROIfinder module, Related to Figure 1. (**A**) Image at 5x magnification with embryos. (**B**) Edge detection. (**C**) Detected ROI, shown as black object masks.

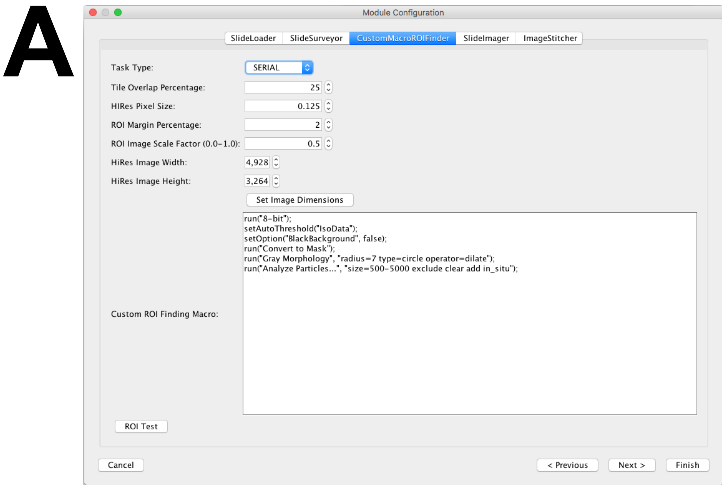

B

```
// SlideSurveyor PostProcessing
run("Bandpass Filter...", "filter_large=25 filter_small=15 suppress=Vertical tolerance=5 autoscale saturate");
run("Bandpass Filter...", "filter_large=25 filter_small=15 suppress=Horizontal tolerance=5 autoscale saturate");

// resize 0.5
run("Scale...", "x=0.5 y=0.5 width=4329 height=3629 interpolation=Bilinear average create");

// CustomMacroROIFinder Custom ROI Finding Macro
run("8-bit");
setAutoThreshold("IsoData");
setOption("BlackBackground", false);
run("Convert to Mask");
run("Gray Morphology", "radius=7 type=circle operator=dilate");
run("Analyze Particles...", "size=500-5000 exclude clear add in_situ");
```

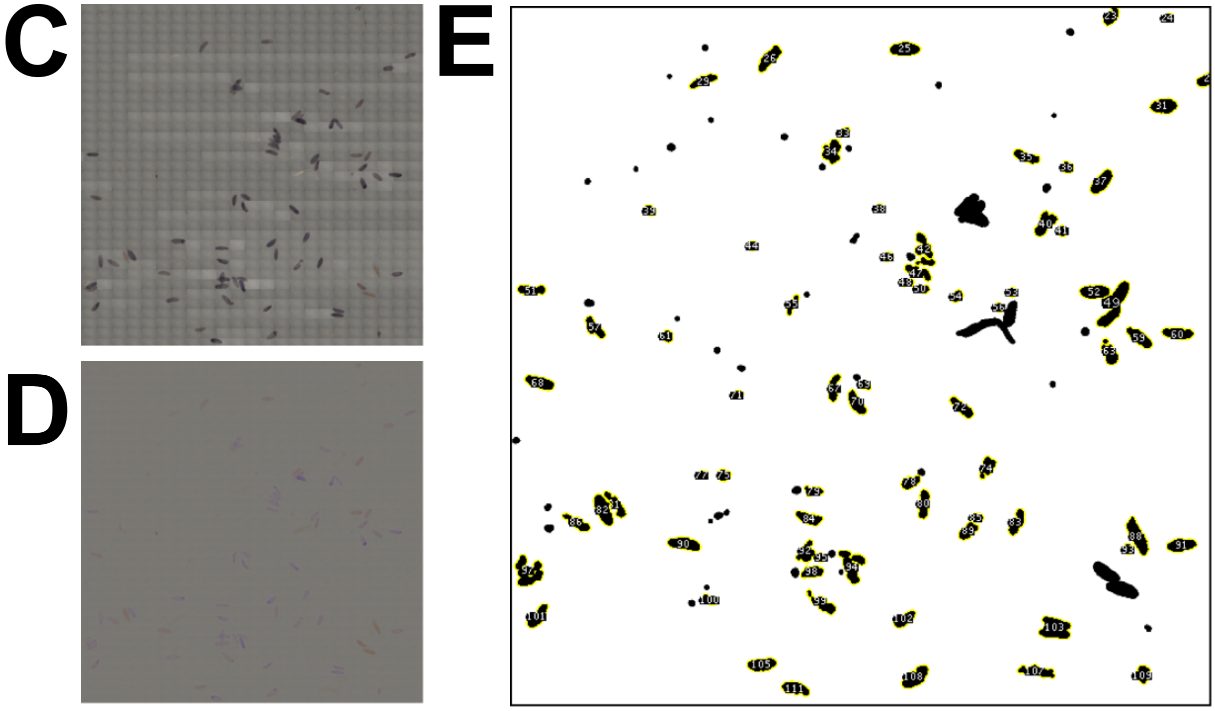

Figure S8. CustomMacroROIFinder for Drosophila imaging, Related to Figure 1. (**A**) Configuration dialog with text box for custom macro. (**B**) Macro used for *Drosophila* imaging workflow. (**C**) Excerpt of the stitched slide from SildeSurveyor. (**D**) Image in panel C after applying FFT Bandpass filter. (**E**) Result after thresholding, morphology and particle detection.

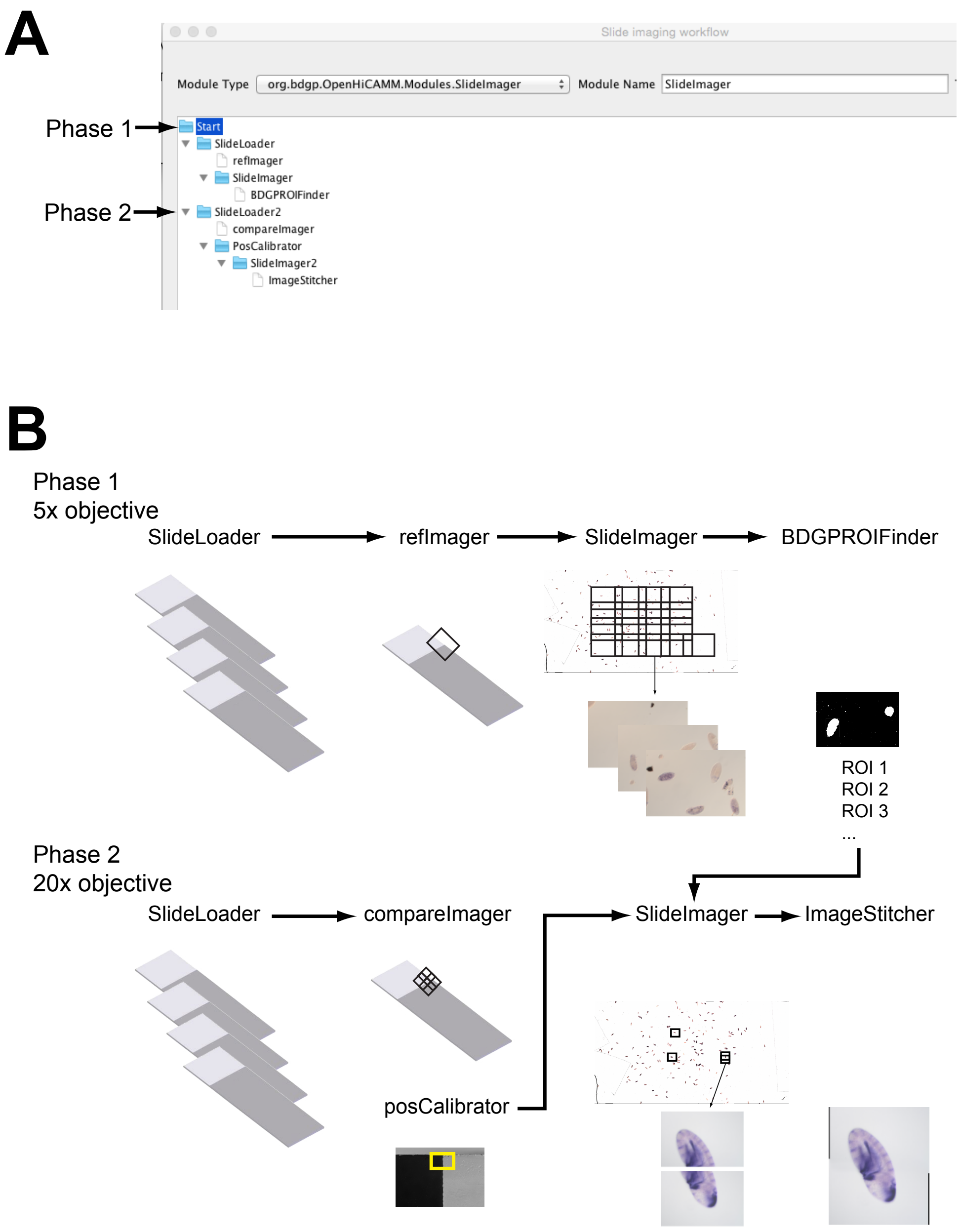

Figure S9. Workflow for Drosophila imaging, Related to Figure 1. Shown is the 5x/20x magnification configuration used for imaging Drosophila embryos. (**A**) Configuration dialog with the workflow arranged in a tree-like structure. Child modules display a higher level of indentation than the parent modules. The workflow execution starts at Phase 1 or 2 entry points and stops when it reaches a point with no further child modules. (**B**) Schematic representation of the workflow. Phase 1 of the workflow loads a slide (SlideLoader), takes a reference image for calibrating the slide position on the stage (refImager), images the slides in overlapping tiles (SlideImager), detects ROIs and stops. Phase 2, configured as second entry point, loads the same slides a second time (SlideLoader), calibrates the position list by taking images around the reference images and finding a matching position (compareImager), images the ROIs from the first workflow (SlideImager) and, if necessary, stitches adjacent images (SlideStitcher).

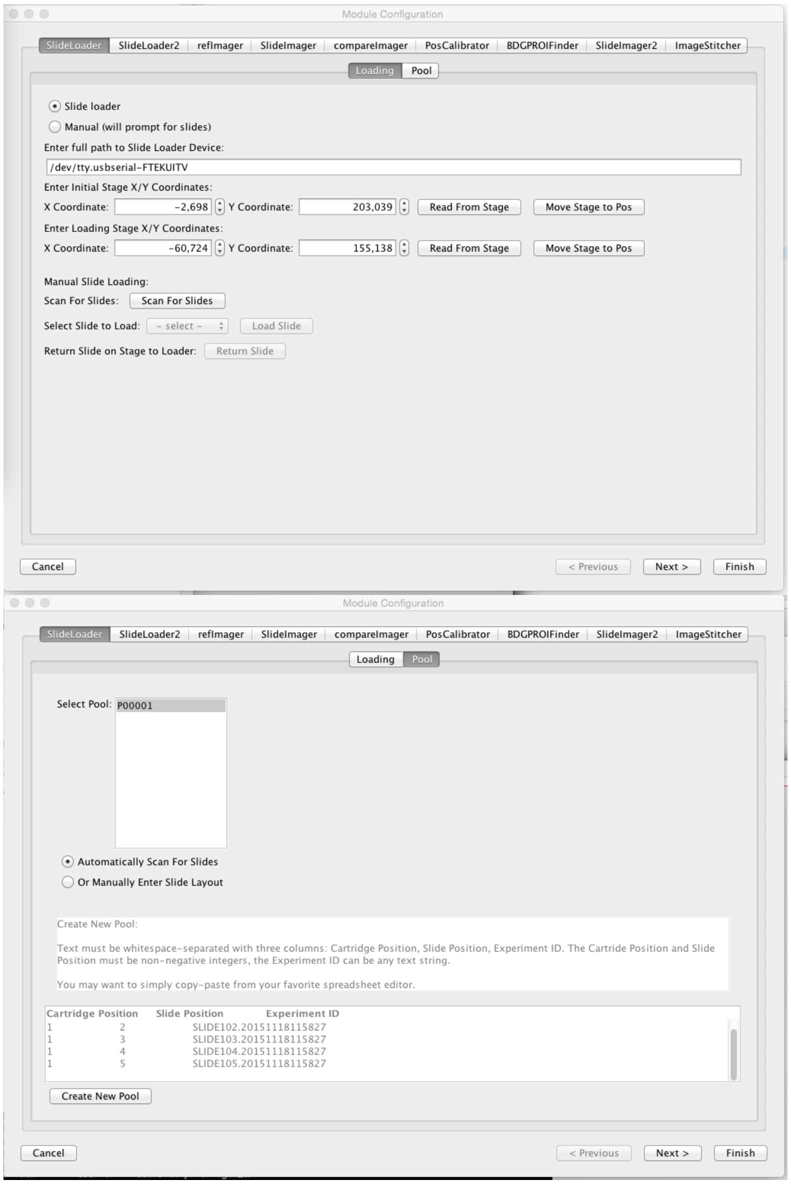

Figure S10. Dialog for module configurations, Related to Figure 1. Configurations for the SlideLoader module are shown. Top of the dialog shows a tabbed list for each module in the workflow, remaining part of the dialog shows the currently selected tab. (**Top**) Basic configuration options for the hardware. For robotic loading of slides, the stage needs to be moved to a position matching the slide loader and the position can be defined here. (**Bottom**) Definition for the metadata of the slide pool. Each slide in the slide loader (a row in the dialog) is located in a cartridge and a position in that cartridge and can be assigned with a descriptive entry.

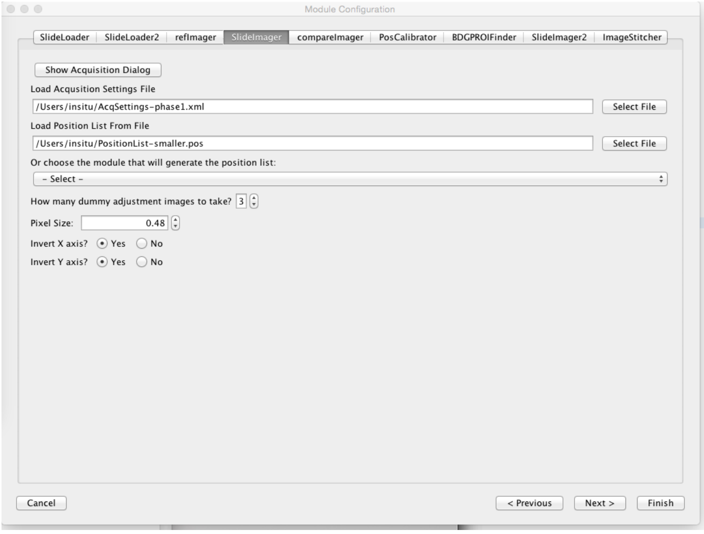

Figure S11. Dialog for module configurations, Related to Figure 1. Shown is the SlideImager. Settings can be configured and saved with the µManager Multi-D dialog (button "Show acquisition dialog"), settings from the Multi-D dialog can be assigned, and a user defined position list or a previously computed position list selected. We found some cameras needed time to adjust to the light. Thus we added a configuration option to take a selectable number of "dummy" images before starting the imaging. The pixel size needs to be calibrated with µManager and entered as configuration to adjust the pixels in the images to the stage position. We also added options to invert the coordinates for the stage compared to the captured image, if necessary.

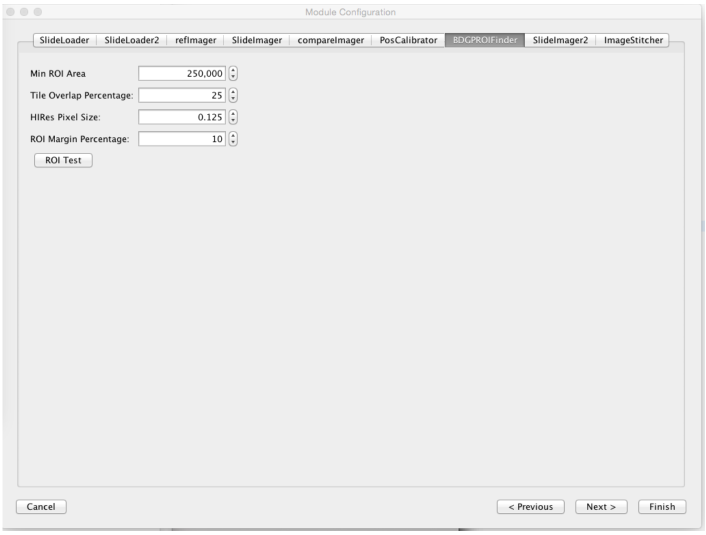

Figure S12. Dialog for module configurations, Related to Figure 1. Shown is our ROIFinder module. The ROIFinder requires a configuration for a minimum size of an object to be recorded (top line), the overlap to adjacent images, the pixel size (Figure G) and a configurable increase for the detected bounding box size.

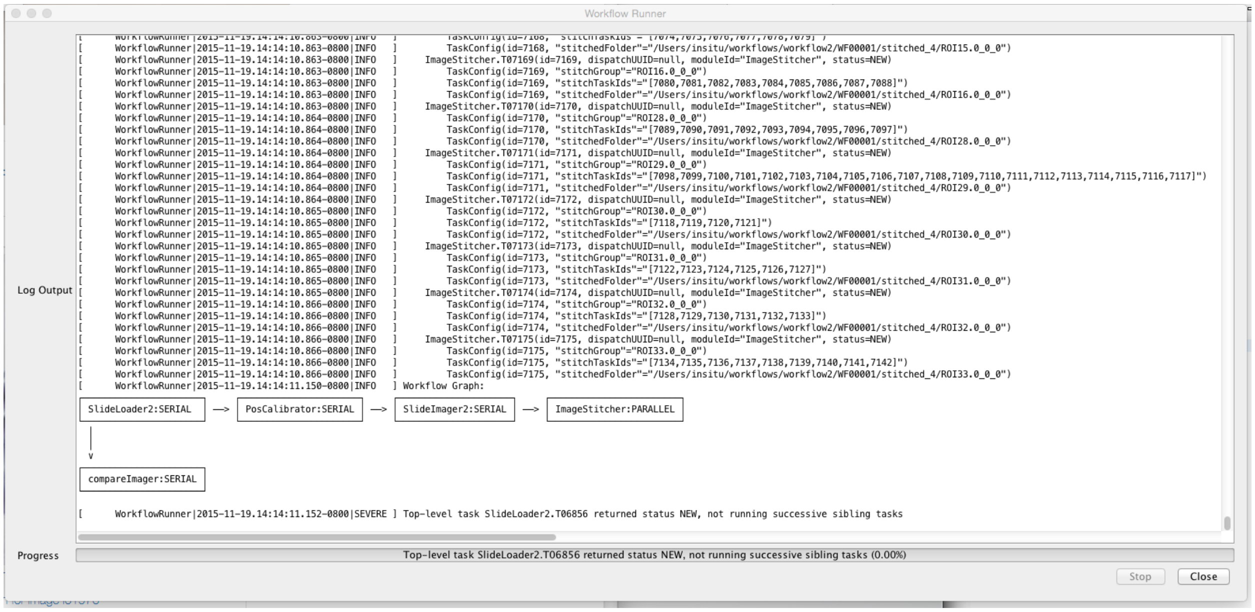

Figure S13. Log output during running the workflow, Related to Figure 1. Initially the workflow manager computes all tasks to complete the currently configured workflow and adds them to the persistent database. The workflow manager displays the currently configured workflow and the tasks that need to be completed sequentially because of hardware dependencies ("SERIAL") or can be executed in parallel ("PARALLEL"), usually only those modules dependent on computations. Progress for each task is printed as log messages, and the overall progress and remaining time shown in a progress bar (bottom).

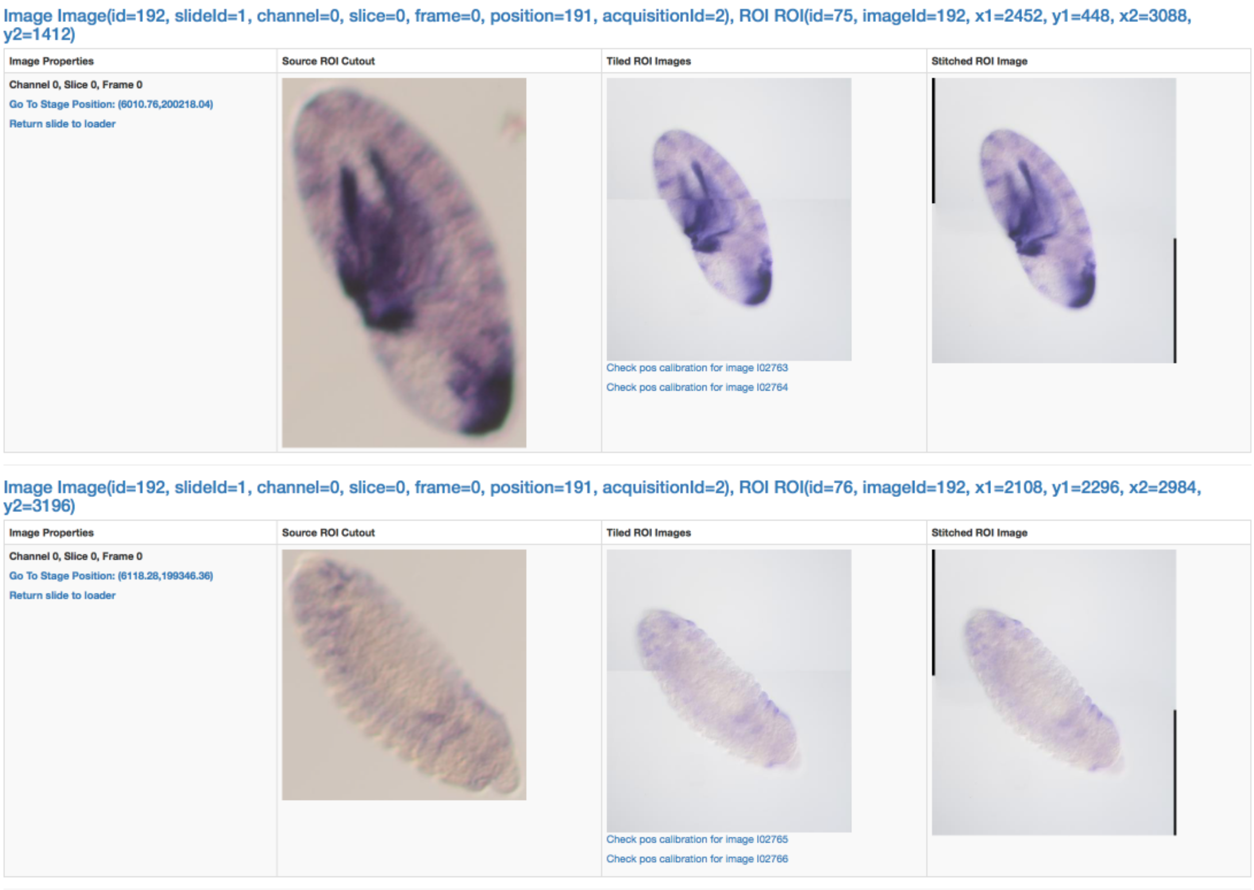

Figure S14. Summary report after acquisition, Related to Figure 1. Shown is a screenshot from the browser. Hyperlinks on the left allow for loading the slide and positioning at the location of the object. The images on the left are from low resolution (5x) phase 1, middle are composite of roughly tiled (according to coordinate position) high resolution phase 2 images (20x), and right composite images showing the result of the image stitching module.

Supplemental Tables

API call	Functionality
initialize	Called during the workflow initialization phase, before module configuration. Allows the module to perform any required initialization.
configure	Called when the user performs the module configuration step. This method returns a Configuration object which handles the configuration UI display, and serialization of configuration options to the database.
createTaskRecords	Creates a database record for each task that is expected to be run during the workflow. Called before the start of the workflow run.
runInitialize	A second initialization entry point. This point is called before each workflow run, whereas initialize is called only once per module.
run	The actual workflow module execution code. Called once per task.
cleanup	Task cleanup code. Is called even if the tasks throws an exception. Called immediately after the run method completes.
setTaskStatusOnResume	Allows customization of the task status when the workflow is run in "Resume" mode.

Table S2. Module API and functions, Related to Figure 1. Custom modules need to implement the shown functions in order to work as OpenHiCAMM workflow module.

API call	Functionality
retrieve	Returns the list of configuration key-value pairs retrieved from the configuration UI
display	When given a list of configuration key-value pairs, display returns a Swing Component that contains the configuration UI, with the configuration applied
validate	Scans the configuration UI for any validation errors, and returns a list of ValidationError objects, which is displayed to the user.

Table S3. Module configuration user interface API and functions, Related to Figure 1.

API call	Functionality
initialize	Pass in the WorkflowRunner context object and perform any required initialization steps.
runReport	Run the report and return the contents of the report as an HTML string.

Table S4. Report interface API and functions, Related to Figure 1.

Supplemental References

Ballard, D. H. (1981). "Generalizing the Hough transform to detect arbitrary shapes." Pattern recognition **13**(2): 111-122.
Pfeiffer, B. D., A. Jenett, A. S. Hammonds, T. T. Ngo, S. Misra, C. Murphy, A. Scully, J. W. Carlson, K. H. Wan, T. R. Laverty, C. Mungall, R. Svirskas, J. T. Kadonaga, C. Q. Doe, M. B. Eisen, S. E. Celniker and G. M. Rubin (2008). "Tools for neuroanatomy and neurogenetics in Drosophila." Proc Natl Acad Sci U S A **105**(28): 9715-9720.
Preibisch, S., S. Saalfeld and P. Tomancak (2009). "Globally optimal stitching of tiled 3D microscopic image acquisitions." Bioinformatics **25**(11): 1463-1465.
Weiszmann, R., A. S. Hammonds and S. E. Celniker (2009). "Determination of gene expression patterns using high-throughput RNA in situ hybridization to whole-mount Drosophila embryos." Nat Protoc **4**(5): 605-618.
Zaharia, M., M. Chowdhury, M. J. Franklin, S. Shenker and I. Stoica (2010). "Spark: Cluster Computing with Working Sets." HotCloud **10**: 10-10.
